# Taxane-Induced Interstitial Lung Disease (Pneumonitis) in Breast Cancer: A Case Series

**DOI:** 10.1159/cro/abzag027

**Published:** 2026-08-19

**Authors:** Jofin George, Srivatsa Lokeshwaran, Sanjana Chetana Shanmukhappa, Tasmiya Kareem, Suhas Dhulipala, Anika Parrikar, Shivaraj Kumar

**Affiliations:** a Department of Pulmonology, Fortis Hospitals, Bangalore, India; b University of Arkansas for Medical Sciences, Little Rock, AR, USA; c Apollo Hospital, Bangalore, India

**Keywords:** Taxanes, Breast cancer, Drug-induced interstitial lung disease

## Abstract

**Introduction:**

Interstitial lung diseases (ILDs) include a variety of conditions characterized by fibrosis and scarring of lung interstitium. Drug-induced ILDs (DILDs) have many clinical patterns, ranging from benign infiltrates to life-threatening acute respiratory distress syndrome.

**Case Presentations:**

Eight cases of patients with breast cancer were clinically diagnosed with ILD after taxane-based chemotherapy. These patients presented with dyspnea, hypoxia, and computed tomography chest findings suggestive of diffuse ground-glass densities suggestive of DILD. These patients were treated with steroids, antibiotics and supportive medications, the offending drug was stopped, and on follow-up showed clinical and radiological clearance.

**Conclusion:**

We highlight the complexities of diagnosing and managing DILD, specifically caused by taxane-class agents. DILD is a diagnosis of exclusion, posing a challenge for clinicians. Early diagnosis, combined with cessation of causative drug and steroid therapy has illustrated better outcomes.

## Introduction

Interstitial lung diseases (ILDs) encompass a heterogenous group of disorders that cause scarring or fibrosis of lung, affecting the pulmonary interstitium [1]. They are broadly categorized into idiopathic, autoimmune-related, and exposure-related [2]. The most prevalent form being idiopathic pulmonary fibrosis, which has a poor prognosis with a median survival of 3–5 years, if left untreated.

Exposure-related ILDs may occur from irritants, radiation therapy or due to drugs. Drug-induced ILD (DILD) can result from lung injury due to chemotherapy, certain cardiac medications, and anti-inflammatory medications. The pathophysiology of DILD may involve direct toxicity to lung tissue, allergic reactions, or triggering of the autoimmune response, leading to interstitial inflammation and eventually fibrosis [3, 4]. Diagnosis of DILD requires a sequential correlation between drug exposure and symptom onset, clinical improvement upon withdrawal of the suspected agent, in addition to clinical and radiological findings consistent with ILD. Most studies reported chemotherapeutic drugs to be the primary cause of DILD, accounting for 23–51% of cases [3]. Other common causes included antibiotics (6–26%), psychiatric medications (0–9%), antiarrhythmic agents (0–9%), non-steroidal anti-inflammatory drugs (0–23%), and disease-modifying anti-rheumatic drugs (6–72%) [4].

In this case series, we present eight instances of DILD induced by taxane-class chemotherapeutic agents. Taxanes include paclitaxel, docetaxel, cabazitaxel, and nanoparticle albumin-bound paclitaxel (nabpaclitaxel), which are anti-microtubule drugs with a broad range of antitumor activity. In this case series, we aim to delineate the clinical presentation, diagnostic criteria, and management strategies for DILD in patients receiving taxane-based chemotherapy.

### Case 1

A 31-year-old woman presented with a lump in the left breast. It was detected as stage IIA carcinoma of the breast(pT1cN1a). Immunohistochemistry revealed triple-negative phenotype with high Ki-67 index (90%). Later, she underwent upfront breast conservation surgery and received 4 cycles of doxorubicin-cyclophosphamide, followed by 11 weeks of paclitaxel, with the last cycle of paclitaxel being omitted due to the development of grade 3 peripheral neuropathy. A month later, the patient presented with dyspnea, oxygen desaturation and multiple febrile episodes, with bilateral crackles on auscultation. Computed tomography (CT) Chest highlighted diffuse ground-glass densities in bilateral lung fields and peribronchial interstitial thickening in the upper lobes. Bronchial smears and cultures were negative for infection. The patient was initially started on intravenous pulse steroids and antibiotics. She also received oxygen supplementation and intensive chest physiotherapy. The patient recuperated well and was discharged after 10 days of admission. Follow-up chest CT indicated resolution of ground-glass densities and interstitial thickening, indicating improvement.

### Case 2

A 63-year-old woman presented with grade 3 invasive carcinoma (pT2N0Mx) of the left breast (ER+/PR+/HER2NEU−). She underwent a left modified radical mastectomy followed by adjuvant chemotherapy. Chemotherapy with 4 cycles of doxorubicin-cyclophosphamide was started, followed by 4 cycles of weekly paclitaxel. On day 1 of the second cycle, the patient complained of dyspnea on exertion, with bilateral crackles on auscultation. Chest X-ray demonstrated diffuse ground-glass opacities in bilateral lung fields. Further evaluation with a CT chest corroborated these findings and additionally showed mild reticular interstitial thickening. Bronchoalveolar lavage showed inflammatory cells and gram-positive cocci on gram smears. The absolute neutrophil count (ANC) was elevated at 21.42 K/µL. Following a week of treatment with intravenous steroids, oral antibiotics and supportive care the patient’s respiratory symptoms improved. She was discharged with continued radiation therapy.

### Case 3

A 47-year-old woman was diagnosed with locally advanced carcinoma of the right breast (ER+/PR+/HER2NEU−), following which, she underwent a right-modified mastectomy and paclitaxel chemotherapy. Within 1 month, the patient developed dyspnea, fatigue and fever. Systemic examination revealed bilateral crackles with an elevated ANC of 13.29 K/µL. Arterial blood gas analysis indicated reduced partial pressure of oxygen. Further, CT chest demonstrated extensive ground-glass densities in bilateral lung parenchyma, with bat-wing pattern involvement and peripheral subpleural sparing. Along with supportive care, the patient received intravenous methylprednisolone, intravenous antibiotics, and bronchodilators. Post-treatment, the patient's condition improved, showing hemodynamic stability and oxygen saturation of 98%. She was discharged 10 days after admission and subsequently underwent radiation therapy.

### Case 4

A 51-year-old woman was diagnosed with stage IV (T2N1M1) carcinoma of the left breast (ER+/PR−/HER2NEU+). Chemotherapy was started with 3 cycles of trastuzumab, pertuzumab, and docetaxel. Two months later, the patient experienced dyspnea, cough and febrile episodes. A CT chest demonstrated ground-glass densities, patches of parenchymal peripheral opacities, and interstitial thickening in basal segments of lower lobes that led to a diagnosis of drug-mediated pneumonitis. Her ANC was 3.40 L/µL. Following intravenous methylprednisolone and supportive care, the patient showed symptomatic improvement and was discharged 10 days after admission. The patient subsequently underwent hormonal therapy.

### Case 5

A 44-year-old woman was diagnosed with carcinoma of the left breast (T2N1M0) (ER+/PR+/HER2NEU−) and underwent breast conservation surgery along with axillary lymph node dissection. Postoperatively, the patient received a dose-dense chemotherapy regimen of 4 cycles of doxorubicin-cyclophosphamide, followed by 12 weeks of paclitaxel. A month later, the patient presented with complaints of cough and intermittent fever. CT chest revealed ground-glass densities with patchy consolidation, and interstitial thickening, leading to the diagnosis of paclitaxel-induced pneumonitis. The patient was treated with an intravenous antibiotics, steroids, and supportive care. Following symptomatic improvement, she was discharged after a week of admission. Radiation therapy was subsequently initiated.

### Case 6

A 56-year-old female underwent neoadjuvant chemotherapy for stage III (T4bN2bM0) carcinoma (ER+/PR+/HER2NEU−, Ki-67 30%) of the right breast. The chemotherapy regimen consisted of 4 cycles of doxorubicin-cyclophosphamide followed by 6 cycles of weekly paclitaxel. One month post chemotherapy, the patient complained of dyspnea on exertion, cough, and fever spikes. CT chest revealed diffuse ground-glass densities in bilateral lung fields with peribronchial atelectasis in bilateral lower and right middle lobes. The patient was treated with intravenous methylprednisolone, intravenous antibiotics, bronchodilators, and supportive care. The patient gradually recovered and was discharged 5 days after admission.

### Case 7

A 39-year-old female, case of stage IIB (cT2N1M0) carcinoma of right breast (ER+/PR+/HER2NEU−) underwent 4 cycles of chemotherapy with epirubicin-cyclophosphamide, followed by one cycle of paclitaxel. Two weeks post paclitaxel, she presented with breathlessness and hypoxia. CT chest showed patchy consolidation and ground-glass opacities in bilateral upper and lower lobes with overlying pleural thickening. Since both bacterial and viral screens were negative, paclitaxel-induced ILD was suspected. She was treated with oxygen support, and tapering doses of prednisone for 8 weeks. Over the course of treatment, her oxygenation improved, and CT also showed resolution of the GGOs. Subsequently, her chemotherapy was changed to Docetaxel, with no worsening post starting (Fig. 1a, b).

**Fig. 1.**
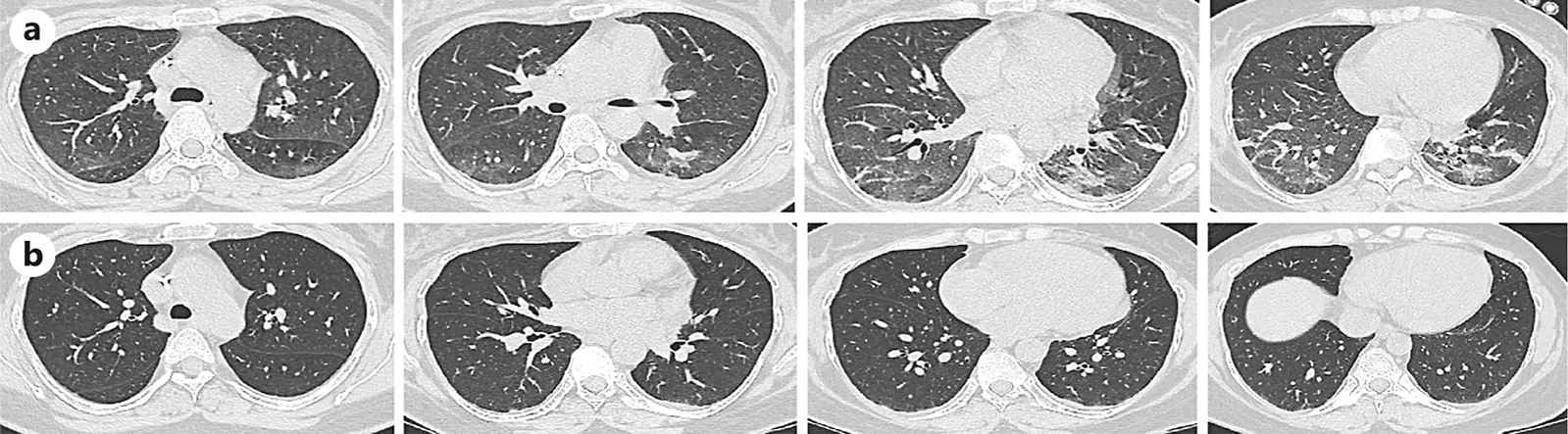
**a** CT Chest showing patchy consolidation and ground-glass opacities in bilateral upper and lower lobes with overlying pleural thickening, following chemotherapy with Paclitaxel in Case 7. **b** Repeat CT after 8 weeks of steroids show resolution of the GGOs in Case 7.

### Case 8

A 56-year-old female, case of carcinoma left breast cT4bN2M0 (ER+/PR+/HER2NEU−) underwent 4 cycles of neoadjuvant chemotherapy with epirubicin-cyclophosphamide, followed by one cycle of paclitaxel. Within 2 weeks, she presented with dyspnea and desaturation on room air. CT chest showed diffuse ground-glass opacities and consolidations predominantly involving the upper lobes, along with scattered centrilobular nodules and mild bronchial wall thickening, suggestive of diffuse pneumonitis. After all infective etiologies were ruled out, a diagnosis of chemotherapy induced pneumonitis was made, and she was started on pulse methylprednisolone for 5 days. Over the course of hospital stay, her oxygenation improved, and she was discharged on 1 week of oral steroids. Repeat CT after 2 weeks showed a near complete resolution of the lung opacities. She later underwent surgery for the same (Fig. 2a, b). Table 1 gives a summary of all cases.

**Fig. 2.**
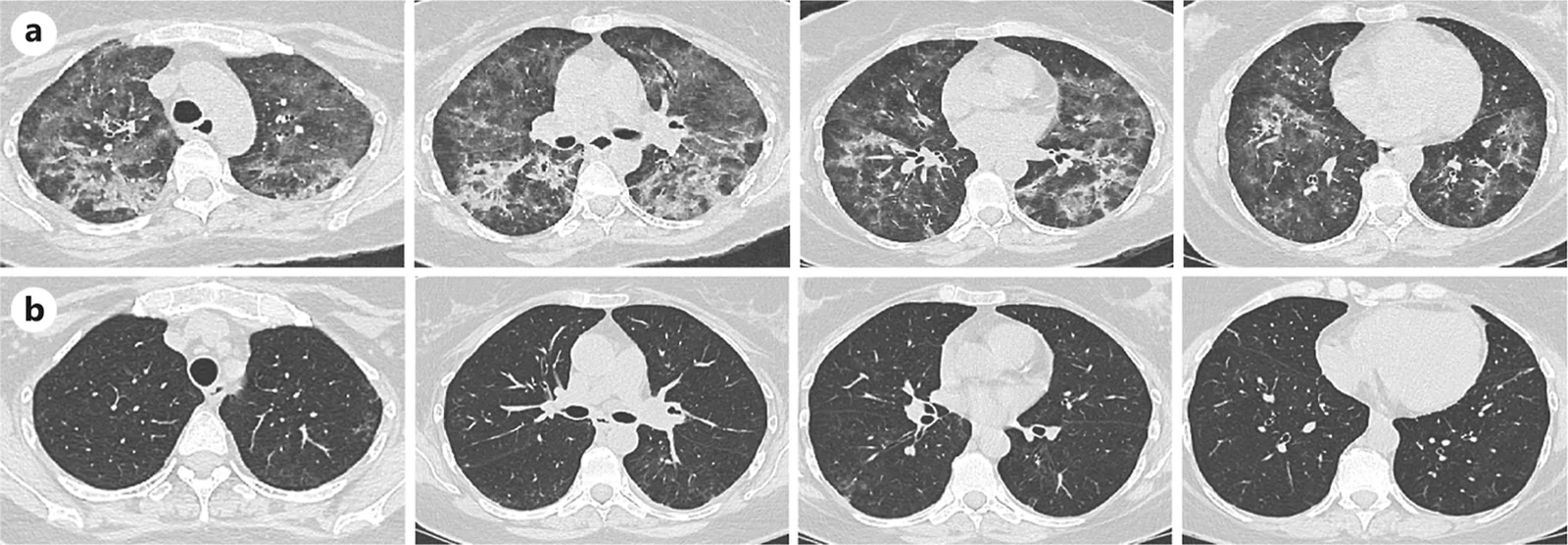
**a** CT Thorax showing diffuse ground-glass opacities and consolidations predominantly involving the upper lobes, along with scattered centrilobular nodules and mild bronchial wall thickening, suggestive of diffuse pneumonitis, following Paclitaxel chemotherapy in Case 8. **b** Repeat CT after 2 weeks of steroid therapy showed a near complete resolution of the lung opacities in Case 8.

**Table 1.** Summary of all cases

Case No.:	Stage and hormonal status	Chemotherapy received	Clinical features	Radiological findings	Treatment
1	pT1cN1a, ER−/PR−/HER2NEU−	Doxorubicin-cyclophosphamide, Paclitaxel	Dyspnea, Hypoxia, Bilateral crackles	Bilateral GGOs, Upper lobe peribronchial intersitial thickening	Steroids, antibiotics, oxygen
2	pT2N0Mx, ER+/PR+/HER2NEU−	Doxorubicin-cyclophosphamide, Paclitaxel	Dyspnea, Bilateral crackles	Bilateral diffuse GGOs, Mild reticular interstitial thickening	Steroids, antibiotics
3	Locally advanced, ER+/PR+/HER2NEU−	Paclitaxel	Dyspnea, Hypoxia, fever, Bilateral crackles	Extensive bilateral GGOs, bat-wing involvement with peripheral subpleural sparing	Steroids, antibiotics, bronchodilators
4	T2N1M1, ER+/PR−/HER2NEU+	Trastuzumab, pertuzumab and docetaxel	Dyspnea, cough, fever	Bilateral GGOs, patchy opacities, Lower lobe interstitial thickening	Steroids
5	T2N1M0, ER+/PR+/HER2NEU−	Doxorubicin-cyclophosphamide, Paclitaxel	Cough, fever	Bilateral GGOs, patchy consolidation, interstitial thickening	Steroids, antibiotics
6	T4bN2bM0, ER+/PR+/HER2NEU−	Doxorubicin-cyclophosphamide, Paclitaxel	Dyspnea, cough, fever	Bilateral GGOs, Bilateral lower lobe peribronchial atelectasis	Steroids, antibiotics, bronchodilators
7	cT2N1M0, ER+/PR+/HER2NEU−	Epirubicin-cyclophosphamide, Paclitaxel	Dyspnea, hypoxia	Bilateral GGOs, patchy consolidation, pleural thickening	Steroids
8	cT4bN2M0, ER+/PR+/HER2NEU−	Epirubicin-cyclophosphamide, Paclitaxel	Dyspnea, hypoxia	Bilateral GGOs, consolidation, diffuse pneumonitis	Steroids

## Discussion

DILD is a significant, but often overlooked complication associated with various pharmacotherapies. Diagnosis is complex, considering the varying severity and onset with each drug and inter-patient variability on receiving the same drug [5]. There are no specific tests to establish toxicity of these drugs; however, a compatible clinical pattern with the absence of an alternative diagnosis would be suggestive [6]. Taxanes have broad-ranging efficacy in the treatment of various malignancies, with adverse pulmonary effects like DILD being rare. Taxanes include Paclitaxel, Docetaxel, Cabazitaxel. Paclitaxel is used for ovarian, breast, lung, esophageal carcinomas, Kaposi sarcoma and off-label indications include head and neck cancer, small-cell lung cancer, upper gastrointestinal adenocarcinomas, hormone refractory prostate cancer, non-Hodgkin’s lymphoma, transitional cell carcinoma of the urothelium, and for stage IIB-IV melanoma. Docetaxel is used for the head and neck, breast, prostate, lung, and gastric carcinomas. Cabazitaxel is used for hormone refractory prostate cancer [7].

Though multiple drugs used in the above case series, including anthracyclines, trastuzumab, and cyclophosphamide have been attributed to cause DILD, the typical clinical presentation of cough, dyspnea, and fever, with radiographic features of interstitial pneumonitis including focal and diffuse opacities, septal thickening, patchy consolidations, ground-glass opacities with a negative bacterial and viral screen, and nonspecific bronchoalveolar lavage findings, suggested taxanes to be the offending drug [8]. Additionally, immediate cessation of taxane therapy, along with steroids, antibiotics, and supportive care, resulted in resolution of infiltrates. Summary of all cases are given in Table 1.

The mainstay of management of DILD involves cessation of the offending drug, followed by corticosteroid therapy. Recent studies indicated that nintedanib has shown curative effects in patients with progressing DILD despite the management strategies mentioned above [4]. Since early treatment is known to have better outcomes, a confirmed diagnosis is not always required before initiation of steroid treatment [9]. As seen in our cases, antibiotics, supportive care including oxygen supplementation and intensive chest physiotherapy, have a central role in recovery.

## Conclusion

In conclusion, DILD is a significant complication of chemotherapy. Diagnosing DILD relies on excluding other diseases, posing a challenge for clinicians. Early diagnosis, combined with cessation of causative drug and steroid therapy has illustrated better outcomes. Therefore, it is vital for clinicians to be vigilant about pulmonary toxicity post taxane treatment and initiate early treatment for improved prognosis.

The CARE Checklist has been completed by the authors for this case report, attached as online supplementary Material (for all online suppl. material, see https://doi.org/10.1159/cro/abzag027).

## Statement of Ethics

Written informed consent was obtained from the patients for publication of the details of their medical case and any accompanying images. This retrospective review of patient data did not require ethical approval in accordance with local/national guidelines. Written informed consent was obtained from the patient for publication of this case report and accompanying images.

## Conflict of Interest Statement

The authors have no conflicts of interest to declare.

## Funding Sources

This study was not supported by any sponsor or funder.

## Author Contributions

Conceptualization: J.G., S.L., S.C.S., and A.P.; data acquisition and analysis: all authors; manuscript drafting and revision: J.G., S.C.S., T.K., S.D., and S.K.; interpretation: all authors. All authors reviewed the manuscript.

## Data Availability

All data generated or analyzed during this study are included in this article and its online supplementary material. Further inquiries can be directed to the corresponding author.
